# Prolonged Honeymoon Period in a Thai Patient with Adult-Onset Type 1 Diabetes Mellitus

**DOI:** 10.1155/2021/3511281

**Published:** 2021-09-01

**Authors:** Yotsapon Thewjitcharoen, Ekgaluck Wanothayaroj, Haruethai Jaita, Soontaree Nakasatien, Siriwan Butadej, Ishant Khurana, Scott Maxwell, Assam El-Osta, Waralee Chatchomchuan, Sirinate Krittiyawong, Thep Himathongkam

**Affiliations:** ^1^Diabetes and Thyroid Center, Theptarin Hospital, Bangkok, Thailand; ^2^Epigenetics in Human Health and Disease Laboratory, Department of Diabetes, Central Clinical School, Faculty of Medicine Nursing and Health Sciences, Monash University, Melbourne, Australia; ^3^Hong Kong Institute of Diabetes and Obesity, Prince of Wales Hospital, The Chinese University of Hong Kong, Hong Kong, China; ^4^University College Copenhagen, Faculty of Health, Department of Technology, Biomedical Laboratory Science, Copenhagen, Denmark

## Abstract

**Objective:**

To describe a usual case of adult-onset T1DM with prolonged honeymoon period for more than 5 years.

**Methods:**

Repeated mixed meal stimulation tests for a period of 6–12 months together with monitoring pancreatic autoantibodies and laboratory data were followed following the onset of diagnosis.

**Results:**

We report a 24-year-old Thai patient with T1DM with sustained remission without antidiabetic medication for more than 5 years while maintaining low-carbohydrate intake and regular exercise. Repeated mixed meal stimulation tests for a period of 6–12 months revealed preserved beta-cell functions. Interestingly, repeated pancreatic autoantibodies at 5 years after diagnosis still showed positive anti-GAD, anti-IA2, and anti-ZnT8.

**Conclusion:**

Restored beta-cell function with complete insulin withdrawal in new-onset T1DM has been reported in very few cases with some common factors as in our patient (low-carbohydrate intake with regular exercise). Delaying autoimmune activity by reducing metabolic load in newly diagnosed T1DM might play a role in maintaining the honeymoon period and could lead to an innovative therapeutic option in new-onset T1DM.

## 1. Introduction

Type 1 diabetes mellitus (T1DM) is heterogeneous in its presentation and progression [1]. The rate of beta-cell deterioration varies among individuals. After diagnosis, there may be a period of temporary restoration of beta-cell function that can last up to 1 year called the “honeymoon” period. This phase of remission could be found in about 50% of children diagnosed with T1DM with recent evidence suggesting that the absence of diabetic ketoacidosis (DKA) at initial presentation, short duration of symptoms, older age at presentation, and strenuous exercise could be potential factors [2–5]. Previous studies have shown residual C-peptide secretion is associated with reduced risk of serious hypoglycemic episodes and long-term diabetic complications [6]. Therefore, prolonging the honeymoon period in newly diagnosed T1DM is a topic of intense interest and research in recent years [7].

During the honeymoon phase, exogenous insulin requirement is commonly reduced to below 50% of the initial insulin dose, but complete remission (insulin independence) remains rare. Most studies were done in pediatric subgroup patients, and the standard definition of partial clinical remission identified as the insulin dose-adjusted A1C was introduced in 2009 to allow for universal consensus [8]. The honeymoon period is a critical period for employing innovative treatments targeted at preserving residual beta-cell mass [9]. Recent data also suggest that obesity in individuals with T1DM could accelerate immune-mediated beta-cell destruction at the early stage following diagnosis [10, 11]. Studying the predictive and maintenance factors in this remission phase could improve our understanding in achieving immunotolerance and the potential to reduce, retard, or cure diabetes.

Adult-onset T1DM exhibits certain unique features when compared to childhood and adolescent-onset T1DM including higher beta-cell function at the onset, slower rate of beta-cell deterioration, and lower incidence of pancreatic autoantibodies [12]. Complete insulin withdrawal in new-onset T1DM has been reported in very few cases [13–15]. Herein, we report a case of a 24-year-old Thai patient with T1DM with sustained complete remission without antidiabetic medication for more than 5 years while maintaining low carbohydrate intake and regular exercise. Repeated mixed meal stimulation test (MMST) every 6–12 months showed preserved beta-cell functions. An informed written consent was obtained from the patient for publication of this case report which has been approved by the ethics committee of Theptarin Hospital, Bangkok, Thailand.

## 2. Case Presentation

A 24-year-old Thai male presented with a 6-month history of polyuria, polydipsia, and weight loss of 15 kilograms (baseline body weight 83 kilograms and BMI of 27.8 kg/m^2^) in March 2016. The initial laboratory data showed plasma glucose of 398 mg/dL and glycated hemoglobin (A1C) of 9.3%. No ketonemia was observed. He was diagnosed with stage 3 of type 1 DM based on clinical presentation and positive autoantibodies of protein tyrosine phosphatase (anti-IA2). Antiglutamic acid decarboxylase (anti-GAD) was negative. He did not drink alcohol or smoke and denied using any drugs. He did not have a history of excessive soft drink or overintake of sugar-containing foods. No family history of diabetes was reported. Thyroid function tests and morning plasma cortisol level were normal. Lipid profiles were all in the normal range (total cholesterol, 168 mg/dL; plasma triglyceride, 73 mg/dL; plasma HDL, 68 mg/dL; and plasma LDL, 85 mg/dL). However, euthyroid Hashimoto's thyroiditis was also diagnosed based on his enlarged thyroid gland (30 grams) and positive thyroid autoantibodies. He was started on basal-bolus insulin regimen for only 2 months, and then A1C reversed to 5.9% within that time. Insulin was gradually withdrawn and completely stopped. Oral saxagliptin 5 mg and metformin 1,000 mg was initiated.

**Figure 1:**
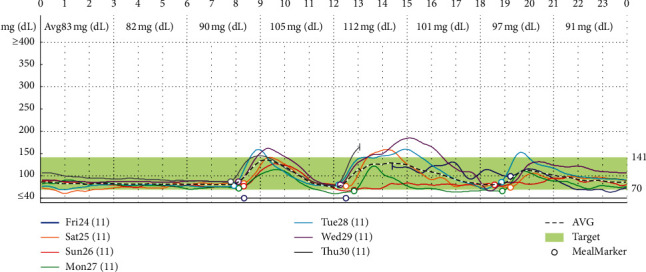
Results of retrospective 6-day continuous glucose monitoring (CGM) system (iPro™2 system, Medtronic MiniMed, Northridge, CA, USA) in the second year (November 2017) following diagnosis revealed normal glucose levels.

**Figure 2:**
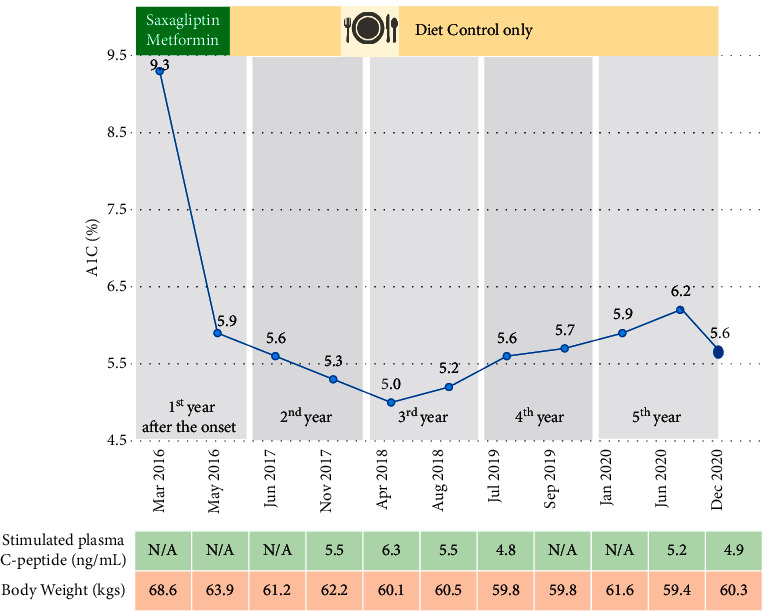
Clinical course and sequential laboratory data in this patient.

He came to Theptarin Hospital in May 2016 to seek second opinion. Next-generation sequencing (NGS) panel for monogenic diabetes including 34 monogenic diabetes-related genes (Table 1 in Supplementary Materials) and a mitochondrial mutation for maternally-inherited deafness and diabetes (MIDD, *mt A3243G*) (GemVCare, Shatin, Hong Kong) revealed negative results. The honeymoon period of T1DM was diagnosed, and he was advised to closely monitor his capillary plasma glucose. Oral antidiabetic medications were tapered within 2 months based on normal glycemic status. In the following months, the patient maintained low-carbohydrate diet (estimated at 90–120 gram per day) and strenuous aerobic exercise to keep his BMI at less than 23 kg/m^2^. He denied any supplemental or herbal medicine usage. Results of retrospective 6-day continuous glucose monitoring (CGM) system (iPro™ 2 system, Medtronic MiniMed, Northridge, CA, USA) in the second year following diagnosis confirmed normoglycemia as shown in Figure 1. MMST was firstly evaluated at the second year after diagnosis to assess the reserved beta-cell function. This procedure was done by the ingestion of 6 mL/kg of Ensure® (Abbott, Illinois, USA) (1 calorie/mL; 65% carbohydrates, 21% protein, and 14% fat) after overnight fasting. Plasma C-peptide was measured by chemiluminescent immunoassay (IMMULITE®, Siemens) which had an interassay coefficient of variation of 3.3% at plasma C-peptide of 0.6 ng/mL. The result revealed preserved beta-cell function with stimulated C-peptide at 5.5 ng/dL. The patient maintains a healthy lifestyle with low-carbohydrate intake and regular exercise 5-6 times per week. His body weight was maintained at 60–63 kilograms (BMI: 20.3–21.3 kg/m^2^) during the past 5-year period. A1C was maintained between 5.0 and 6.2% without any antidiabetic medication for more than 5 years as shown in Figure 2. Serial MMST in every 6–12 months still revealed preserved beta-cell functions.

Pancreatic autoantibodies had also been monitored during follow-up in order to understand ongoing autoimmune status in this unusual patient with T1DM. Interestingly, repeated anti-IA2 at 3 years after diagnosis showed lower titer at 549.4 IU/L and then turned negative in the fourth year after diagnosis. However, anti-IA2 converted to positive again (at the titer of 315.2 IU/L) at the latest follow-up as shown in Table 1. Anti-GAD also converted to positive at low-titer (28.9 IU/mL) in the fifth year after the onset of disease, while anti-ZnT8 was still positive at the latest follow-up. The patient was advised to maintain his bodyweight and healthy behavior together with closely regular OPD follow-ups. At the last follow-up (December 2020), he remained in remission without antidiabetic medication. No ketonuria was detected during follow-up.

## 3. Discussion

This case report highlights the potential for altering the natural history of T1DM in some patients by maintaining low-carbohydrate diet and a healthy lifestyle. To the best of our knowledge, our case represents the longest complete remission from any antidiabetic treatments in newly diagnosed people with T1DM. The honeymoon phase in this patient is sustained for more than 5 years, albeit with ongoing positive pancreatic autoantibodies. The possibility of monogenic diabetes misdiagnosis was also excluded from genetic testing. This immunotolerance phenomenon is an example of metabolic flexibility and immune escape [16]. Currently, ongoing clinical trials attempt to prevent or slow disease progression by targeting immune system. However, progress and achievement remain limited.

**Figure 3:**
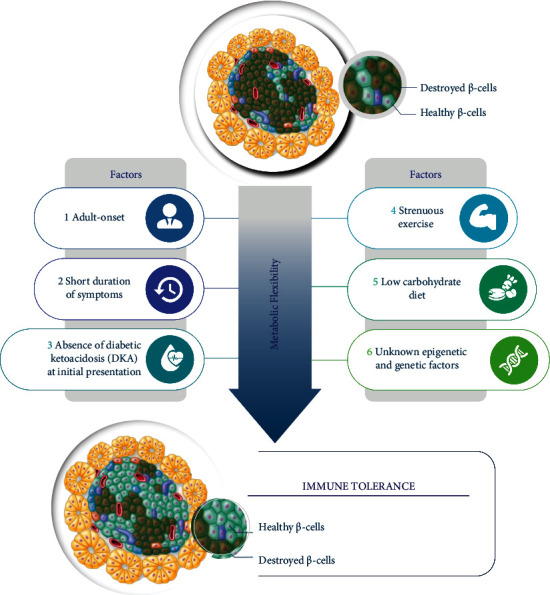
Postulated mechanisms of prolonged honeymoon period in this patient.

Several other features present in this patient might also contribute to this exceptional clinical course as summarized in Figure 3. Age at the onset is a major determinant of beta-cell deterioration. Previous studies consistently confirmed that children with severe diabetic ketoacidosis at the time of diagnosis are less likely to enter a partial remission phase, reflecting more progressive and rapid destruction of beta-cell function [2–7]. During the honeymoon period, re-establishment of self-tolerance has also been shown to be influenced by modifiable factors. Low-carbohydrate diet (less than 150 gram per day) and diet with low *n *− 6/*n *− 3 essential fatty acid ratio could prolong survival of experimental mice model with T1DM [17, 18]. Also, higher level of exercise in men with newly diagnosed T1DM showed the benefits in the extension of the honeymoon period and is five times longer when compared to sedentary men [19]. Therefore, the benefits of maintaining a healthy lifestyle following the diagnosis of T1DM should be investigated in more detail as a potential modifiable factor to delay beta-cell destruction. Nevertheless, it should be emphasized that carbohydrate should not be excessively restricted (less than 60 grams per day) in children and adolescents with T1DM given the increasing risk of eating disorders [20].

Although partial remission is well documented in T1DM, complete insulin withdrawal has been rarely reported. In reviewing literature, we found only four cases from 3 reports with complete remission of more than 1 year following the diagnosis of T1DM [13–15]. Three of the four cases were treated with oral sitagliptin as an off-label treatment. Our present case was also treated with dipeptidyl peptidase 4 (DPP-4) inhibitor (saxagliptin) for 2 months. Dietary factors and physical activity following the diagnosis of T1DM rather than initiating DPP4i in this patient might be a major factor to improve resilience toward autoimmunity. The potential immunoregulatory effects of this class of oral antidiabetic medication have been also reported in patients with latent autoimmune diabetes in adults (LADA) [21, 22]. Despite convincing clinical results using DPP-4 inhibitors to improve beta-cell function and attenuate autoimmunity in LADA, there are several differences between LADA and T1DM [23]. Further clinical studies are needed to understand the role of DPP-4 inhibitor as a disease-modifying medication in T1DM. Clinical studies on GLP-1 receptor agonists suggest possible benefit on the improvement of postprandial hyperglycemia and reduced insulin requirement in patients with T1DM. These effects were attributed to the effect of GLP-1 on gastric emptying and glucagon suppression, rather than a direct effect C-peptide release [24]. Other noninsulin adjunctive therapies for T1DM had also been extensively studied but yielded only small benefits [25].

The role of genetic and/or epigenetic factors that might influence the honeymoon phase remains poorly understood. Preclinical studies have shown changes in DNA methylation of beta-cell-specific CD8+ T cells related with the longevity of human autoreactive T-cell responses and the rate of T1DM progression [26], and this is consistent with recent studies showing immunological changes in peripheral T cells [27]. Manipulating epigenetic mechanisms in these autoreactive T cells might be one of the potential targets to re-establish self-tolerance.

## 4. Summary

We report an unusual case of T1DM with sustained remission of more than 5 years while a maintaining low-carbohydrate intake with regular exercise. Delaying autoimmune activity by reducing metabolic load in newly diagnosed T1DM might play a role in extending the honeymoon period together with unknown genetic and/or epigenetic factors. Longer duration of follow-up in this patient and further detailed studies in the mechanism of immune tolerance should provide an opportunity to develop innovative targets for curing or improving the prognosis of T1DM.

## Figures and Tables

**Table 1:** Sequential pancreatic autoantibodies from the initial diagnosis to the fifth year of the development of T1DM.

	At the time of diagnosis (march 2016)	3^rd^ year after the diagnosis (September 2018)	4^th^ year after the diagnosis (January 2019)	5^th^ year after the diagnosis (December 2020)
Anti-GAD (<5 IU/mL)	Negative	N/A	Negative	28.9
Anti-IA2 (<5 IU/mL)	>2,000	549.4	<7.5	315.2
Anti-ZnT8 (<15 IU/mL)	N/A	N/A	948	707.8

N/A: not available.

## Data Availability

The data used to support the findings of this study are available from the corresponding author upon request.

## Supplementary Materials

Supplementary Materials *Table 1*. The panel consists of known genes for monogenic diabetes including all exomes and intron-exon junction detected by next-generation sequencing. The selection of genes was based on Ellard S et al. (Diabetologia 2013;56:1958–6) and previously published results.
