# Supplementary material for: Prolonged Honeymoon Period in a Thai Patient with Adult-Onset Type 1 Diabetes Mellitus
Source: Case Rep Endocrinol. 2021 Sep 1;2021:3511281. doi: 10.1155/2021/3511281 (PMC8429022; doi:10.1155/2021/3511281)
Supplement: Supplementary Materials — Table 1. The panel consists of known genes for monogenic diabetes including all exomes and intron-exon junction detected by next-generation sequencing. The selection of genes was based on Ellard S et al. (Diabetologia 2013;56:1958–6) and previously published results. [file 3511281.f1.docx]

**Supplementary material**

**Table 1**. The panel consists of known genes for monogenic diabetes including all exomes and intron-exon junction detected by next generation sequencing. The selection of genes was based on Ellard S, et al. (Diabetologia 2013;56:1958-6) and previously published results.

| *ABCC8* | ATP-binding cassette transporter sub-family C member 8 |
| --- | --- |
| *AKT2* | AKT Serine/Threonine Kinase 2 |
| *APPL1* | Adaptor Protein, Phosphotyrosine Interacting With PH Domain And Leucine Zipper 1 |
| *CEL* | Carboxyl ester lipase |
| *CISD2* | CDGSH Iron Sulfur Domain 2 |
| *DCAF17* | DDB1 And CUL4 Associated Factor 17 |
| *DNAJC3* | DnaJ Heat Shock Protein Family (Hsp40) Member C3 |
| *DYRK1B* | Dual Specificity Tyrosine Phosphorylation Regulated Kinase 1B |
| *GATA4* | GATA Binding Protein 4 |
| *GATA6* | GATA Binding Protein 6 |
| *GCK* | Glucokinase |
| *HNF1A* | Hepatocyte nuclear factor-1 alpha |
| *HNF1B* | Hepatocyte nuclear factor-1 beta |
| *HNF4A* | Hepatocyte nuclear factor-4 alpha |
| *INS* | Insulin |
| *INSR* | Insulin receptor |
| *KCNJ11* | Potassium Inwardly Rectifying Channel Subfamily J Member 11 |
| *LMNA* | Lamin A/C |
| *NEUROD1* | Neurogenic differentiation 1 |
| *PAX4* | Paired box gene 4 |
| *PAX6* | Paired box gene 6 |
| *PCBD1* | Pterin-4 Alpha-Carbinolamine Dehydratase 1 |
| *PDX1* | Pancreatic And Duodenal Homeobox 1 |
| *PIK3R1* | Phosphoinositide-3-Kinase Regulatory Subunit 1 |
| *PLIN1* | Perilipin 1 |
| *POLD1* | DNA Polymerase Delta 1 |
| *PPARG* | Peroxisome proliferator-activated receptor |
| *PPP1R15B* | Protein Phosphatase 1 Regulatory Subunit 15B |
| *RFX6* | Regulatory factor X6 |
| *SLC29A3* | Solute Carrier Family 29 Member 3 |
| *TRMT10A* | TRNA Methyltransferase 10A |
| *WFS1* | Wolframin ER Transmembrane Glycoprotein |
| *ZBTB20* | Zinc Finger And BTB Domain Containing 20 |
| *ZFP57* | Zinc finger protein 57 homolog |
